# Bi-stability in femtosecond laser ablation by MHz bursts

**DOI:** 10.1038/s41598-024-54928-7

**Published:** 2024-03-07

**Authors:** Andrius Žemaitis, Mantas Gaidys, Paulius Gečys, Mindaugas Gedvilas

**Affiliations:** Department of Laser Technologies (LTS), Center for Physical Sciences and Technology (FTMC), Savanorių Ave. 231, 02300 Vilnius, Lithuania

**Keywords:** Theory and computation, Computational methods, Ultrafast lasers, Surface patterning, Design, synthesis and processing, Laser-produced plasmas

## Abstract

In this work, a bi-stable behavior of laser ablation efficiency and quality was controlled by fluence and burst length. The plasma shielding of incoming laser radiation caused sudden jumps with a significant decrease in ablation efficiency for every even number of pulses in the burst. The attenuation of incoming laser radiation by plasma created by the previous pulse was incorporated into the toy model of burst ablation efficiency. The mathematical recurrence relation has been derived for the first time, binding ablation efficiency for the next pulse with the efficiency of the previous pulse, which predicts bi-stability, as well as sudden jumps occurring in ablation efficiency depending on the number of pulses in burst with the response to changes of the control parameter of peak laser fluence in the pulse. The modeling results using new recurrence relation showed stable and bi-stable ablation efficiency depending on burst fluence and the number of pulses, which agreed well with experimental data. The extremely efficient laser ablation has been achieved by optimizing the shielding effect using three pulses in the burst.

## Introduction

Ultra-short laser pulses have been applied for precision micro/nano-fabrication for metals, semiconductors, insulators, and biological materials for science^1^, technology^2^, industry^3^, and medicine^4^. The foundation of the ablation cooling effect by GHz bursts of femtosecond pulses has made a huge impact on the real industrial application of laser machining because of the enhancement of the ablation rate and minimization of thermal damage by an order of magnitude^5^. The burst mode irradiation is used in a wide range of pulse repetition rates from MHz^6–9^, to GHz^7–12^, and even THz^13–15^. However, a big difference in ablation efficiency has been reported depending on the parity of integer pulse number (odd or even) in the burst^6,7,11,13,16,17^. In majority of the reports, the shielding or/and re-deposition are indicated as the main reasons resulting in bistable dependence of ablation efficiency on the pulse number in the burst. The redeposition hypothesis by second sub-pulse for copper has investigated and verified by atomistic simulations in^18^. The re-deposition has been experimentally studied by shadowgraph technique; however, no clear experimental evidence of re-deposited material was found^17^. The shielding effect has been identified as the dominating effect resulting drop in ablation efficiency for the second sub-pulse in time-resolved experiments for copper^19^. It has been shown experimentally that incoming irradiation is attenuated by ejected particles, vapor, and plasma of the previous pulse, thus reduced ablation efficiency is observed for even pulses^13,20,21^. For odd pulses, higher efficiency is observed, because the previous even pulse produces fewer ablation products and more incoming laser radiation reaches the sample^6^. This leads to bi-stable ablation efficiency, however, there is no paper in scientific literature dedicated to the analytical/numerical modeling of the shielding effect influence on the ablation efficiency and stability/bi-stability analysis. Moreover, in the vast majority of experimental research works the bi-stable behavior of ablation efficiency depending on burst length was investigated for the single burst fluence^6,7,11,13,16,17^, and there is no scientific paper dedicated to efficient bi-stability effect investigation dependence on bust fluence and length, simultaneously.

In this study, we present new experimental and theoretical results on the bi-stability control of ablation efficiency for MHz bursts of femtosecond laser pulses. The experiments were conducted to measure laser ablation efficiency using a femtosecond laser working in MHz burst mode. The experimental results showed stable and bi-stable ablation efficiency regions depending on the burst fluence and the number of pulses in a burst. The shielding effect by the cloud of ablation products (particles, vapor, and plasma) was included in the toy model of laser ablation efficiency of parabolic dimple for the first time. The analytical expression mathematically called recurrence relation has been found, which combines ablation efficiency for the next pulse with the efficiency of the previous pulse. Nonlinear recurrence relation predicts stable and bi-stabile ablation, as well as sudden jumps in ablation efficiency depending on burst fluence and the parity of an integer number of pulses in the burst. The equations of the toy model were numerically solved, and computational results showed bifurcation in ablation efficiency, which coincided well with experimental data. To the best of our knowledge, we have exceeded the highest laser milling efficiency for copper recorded in the scientific literature^11^.

## Experiment

### Laser milling setup

A solid-state femtosecond burst laser (Pharos, Light Conversion) with a pulse duration of *τ*_FWHM_ = 210 fs, a central wavelength of *λ* = 1030 nm, the burst repetition rate of *f*_burst_ = 100 kHz, and an average laser power output of *P* = 7.3 W was used in experiments. The number of pulses per bust number was controlled from *N* = 1 (single pulse regime) to *N* = 9. The intra-burst pulse repetition rate was *f*_pulse_ = 64.7 MHz, which determined the temporal distance between pulses of *t*_pulse_ = 15.5 ns.

**Figure 1:**
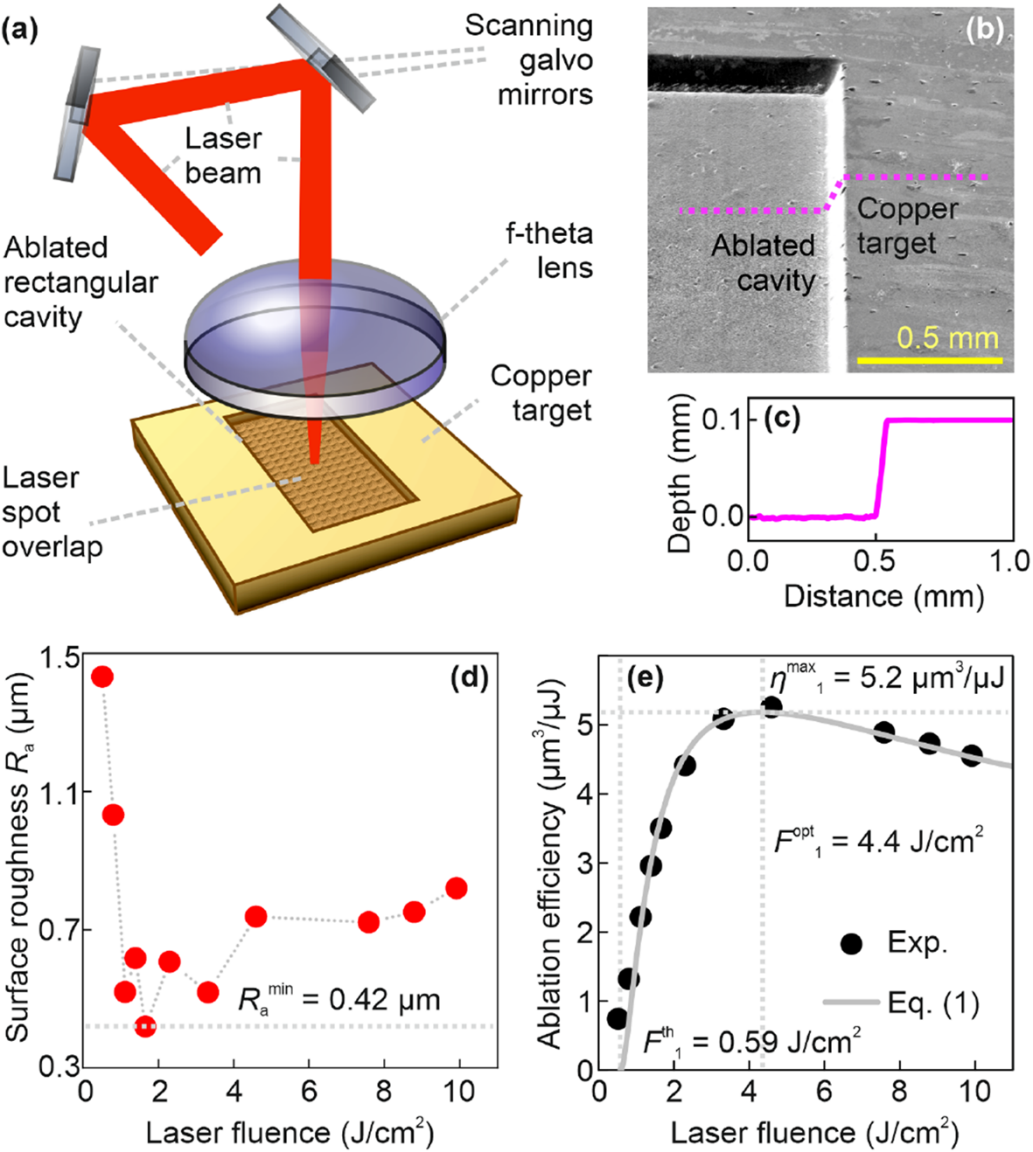
Laser ablation efficiency experiment using femtosecond single pulses. (**a**) Principal experimental setup: the position of the incoming laser beam on the copper target surface is controlled by two galvanometer-based scanning motors with mounted optical mirrors, the beam is focused using the telecentric f-theta lens, and a rectangular cavity is ablated in copper by bi-directional laser beam scanning which produces overlapped laser spots on the ablated cavity bottom. (**b**) SEM image of the rectangular-shaped cavity in copper ablated by using femtosecond pulses, cavity dimensions of 2.0 × 1.0 × 0.1 mm^3^, the image was taken at a tilt angle of 60°. (**c**) Profile of laser ablated cavity, average depth 100 ± 5 μm of the rectangular cavity. (**d**) Surface roughness *R*_a_ of laser ablated cavity bottom and (**e**) ablation efficiency of copper dependence on laser fluence by conventional single pulse irradiation mode. The number of pulses in the burst *N* = 1 (single pulse regime), pulse duration *τ*_FWHM_ = 210 fs, laser wavelength *λ* = 1030 nm, pulse repetition rate *f*_pulse_ = *f*_burst_ = 100 kHz, dots—experimental data points, line–fit by Eq. (1), fit parameters *F*^th^_1_ = 0.59 ± 0.02 J/cm^2^, *δ* = 113 ± 5 nm.

A galvanometer scanner (Intelliscan 14, Scanlab) equipped with an f-theta lens with a focal distance of 100 mm was used for beam focusing and positioning on the sample surface (Fig. 1a).

Rectangular-shaped cavities have been ablated by laser into copper with transverse dimensions of 2 mm × 1 mm. The laser processing experiments were conducted at room temperature in air. Several layers ranging from 3 to 21 were ablated to increase the cavity depth up to 100 µm for the higher accuracy of depth profile measurement. The Rayleigh length of the Gaussian beam was 1.2 mm and was more than 10 times larger than the cavity depth. The saturation behavior of ablation depth per layer was never reached and the laser-milled cavity depth had a linear dependence on the number of ablated layers. The lateral distance between bi-directional beam scanning lines of Δ*y* = 10 µm and scanning speed of *v*_x_ = 0.33 m/s, were kept constant during experiments.

### Fluence optimization

There are two competing techniques devoted to maximum laser ablation efficiency via laser fluence optimization: laser fluence variation by adjusting the average laser power at constant spot size and fluence control by varying the laser spot size keeping the highest available laser power^22,23^. Both techniques have advantages and disadvantages. For instance, the variation of laser fluence by changing the focus position on the target is not the best option to compare the results of burst ablation efficiency at different fluences. At small beam waist diameters obtained on target by strong focusing, the plasma expansion is more 3D than 1D, especially at long time scale as in multiple pulse regime. Thereby, the bi-stable behavior and the roughness of the ablated structure could be different in case the fluence would be varied by changing the pulse energy rather than the laser spot diameter on target. Therefore, in this work it would make sense to change pulse energy at the fixed spot radius, keeping similar beam focusing conditions during tests. However, the pulse variation procedure is usually performed with a high-power ultrafast laser with output powers up to 50 W^22–24^, to reach all required fluence values with certain fixed pulse diameters. In this work, we have used a state-of-the-art burst laser with limited output power capabilities of 7.3 W, thus we had limited freedom in selecting the optimization test. Therefore, we used spot diameter optimization instead of pulse energy optimization to achieve optimal fluence for maximal ablation efficiency, which is common optimization at those power levels^25^. In our current ablation efficiency and surface roughness optimization experiments, we always used the maximal available laser power of 7.3 W. Even though, reaching optimal fluence for maximum ablation efficiency was challenging for a larger number of sub-pulses since burst energy is divided by several sub-pulses which drastically decreases peak pulse fluence.

The laser fluence on the sample surface was changed by controlling the *z* position of the sample in respect of the focused laser beam. The *z* position was varied from 0.0 mm (focus position) to 5.3 mm. The spot radius on the sample was characterized by a technique described in^25,26^ and it ranged from 21 to 95 µm. The relative error on average for measured beam radiuses was less than 2.7%. The details of spot radius measurement are given in the Methods section.

### Roughness characterization

Solid copper (CW004A, Ekstremalė) target with dimensions of 50 mm × 50 mm × 5 mm, purity of 99.9%, and roughness of the polished surface of *R*_a_ < 0.1 µm was used in laser milling experiments. For the characterization of surface morphology, the scanning electron microscope (SEM) (JSM-6490LV, JEOL) was employed (Fig. 1b). The depth of laser-milled cavities was measured by using a surface profilometer (Dektak 150, Veeco) (Fig. 1c). In the first part of the research, the surface roughness *R*_a_ of the laser-milled cavity and ablation efficiency dependence on laser fluence was investigated for the conventional single-pulse irradiation regime, with *N* = 1 pulse in the burst. From the measured profiles of the laser-ablated cavity bottom, the surface roughness was determined. The surface roughness can be characterized by different roughness parameters such as *R*_a_, *R*_q_, *R*_*v*_, *R*_p_, *R*_z_, *R*_sk_, *R*_ku_*, R*_tm_, and *R*_z_, however, the arithmetic average of the roughness profile *R*_a_ is by far the most common and mostly used in the scientific literature defining the surface of laser-milled areas^16,23,27–31^. Even though *R*_a_ is mostly used often for historical reasons rather than any particular advantage, as early roughness gauges could only measure surface roughness *R*_a_. The experimentally measured surface roughness *R*_a_ dependence on laser fluence for *N* = 1 pulse in the burst is depicted in (Fig. 1d). The surface roughness *R*_a_ rapidly decreased from 1.43 to 0.42 µm with the increase of laser fluence from 0.52 to 1.66 J/cm^2^, and then the surface roughness *R*_a_ value slowly increased from 0.42 to 0.82 µm with the increase of laser fluence from 1.66 to 9.9 J/cm^2^ (Fig. 1d). The relative error on average for measured surface roughness *R*_a_ and fluence evaluation was less than 2.5% and 3.9%, respectively. The details of surface roughness and fluence evaluation are given in the Methods section.

### Efficiency characterization

From the depth of the laser-ablated cavity measured from the profiles, the ablation efficiency was extracted. The ablation efficiency dependence on laser fluence for *N* = 1 pulse in the burst is depicted in (Fig. 1e). The experimentally measured ablation efficiency increased rapidly from 0.74 to 5.3 µm^3^/µJ with the increase of laser fluence from 0.52 to 4.6 J/cm^2^, and then the ablation efficiency value slowly decreased from 5.3 to 4.6 µm^3^/µJ with the increase of laser fluence from 4.6 to 9.9 J/cm^2^ (Fig. 1e). The relative error on average for measured ablation efficiencies was less than 3.0%. The details of ablation efficiency evaluation are given in the Methods section. The ablation efficiency *η*^pulse^_*n*_ which is equal to ablated volume per pulse *V*_*n*_ divided by the pulse energy *E*_pulse_ in the burst can be expressed by^32–34^:

1 $$\eta_{n}^{{{\text{pulse}}}} = \frac{\delta }{{2F_{{{\text{pulse}}}} }}\ln^{2} \left( {\frac{{F_{n} }}{{F_{n}^{{{\text{th}}}} }}} \right),$$

where *δ* is an effective penetration depth, *F*_pulse_ is the peak laser fluence in the center of the incoming pulse of Gaussian beam *F*_pulse_ = 2*E*_pulse_/(π*w*^2^), *w* is Gaussian beam radius at 1/*e*^2^ level, *n* is the integer number indexing the pulse position in the burst. The *F*_*n*_ is the peak laser fluence that reaches the material. For the first pulse (*n* = 1) fluence at the material surface is equal to the fluence of the incoming beam as *F*_*n*=1_ = *F*_pulse_ because there is no cloud above the material and all incoming fluence reaches the sample, however, for the next pulses (*n* > 1), the peak laser fluence which reaches the material is smaller then incoming fluence as *F*_*n*>1_ < *F*_pulse_, because the incoming pulse is shielded by ablation cloud created by the previous (*n* − 1) pulse. The theoretical optimal fluence for maximal ablation efficiency can be evaluated from the first derivative of Eq. (1) as *F*^opt^_1_ = *e*^2^*F*^th^_1_ ≈ 7.39*F*^th^_1_^32–34^. Also, knowing the optimal fluence the theoretical maximal laser ablation efficiency can be evaluated from Eq. (1) as *η*^max^_1_ = 2*δ*/(*e*^2^*F*^th^_1_) ≈ 0.271 *δ*/*F*^th^_1_. The experimental data were fitted by using Eq. (1) and the fit parameters *F*^th^_1_ = 0.59 ± 0.02 J/cm^2^, *δ* = 113 ± 5 nm were retrieved. The experiment had a good agreement with the ablation efficiency model equation (Fig. 1e) for a single pulse ablation regime (*N* = 1 pulse in the burst). Knowing single pulse ablation threshold, theoretical optimal laser fluence *F*^opt^_1_ = *e*^2^*F*^th^_1_ = 4.4 ± 0.2 J/cm^2^ for the most efficient ablation *η*^max^_1_ = 5.2 ± 0.2 µm^3^/µJ can be found from Eq. (1), which coincides well with experimentally observed optimal laser fluence *F*^opt^_1_ = 4.6 J/cm^2^ for the most efficient ablation *η*^max^_1_ = 5.3 µm^3^/µJ.

## Results and discussion

### Surface roughness

**Figure 2:**
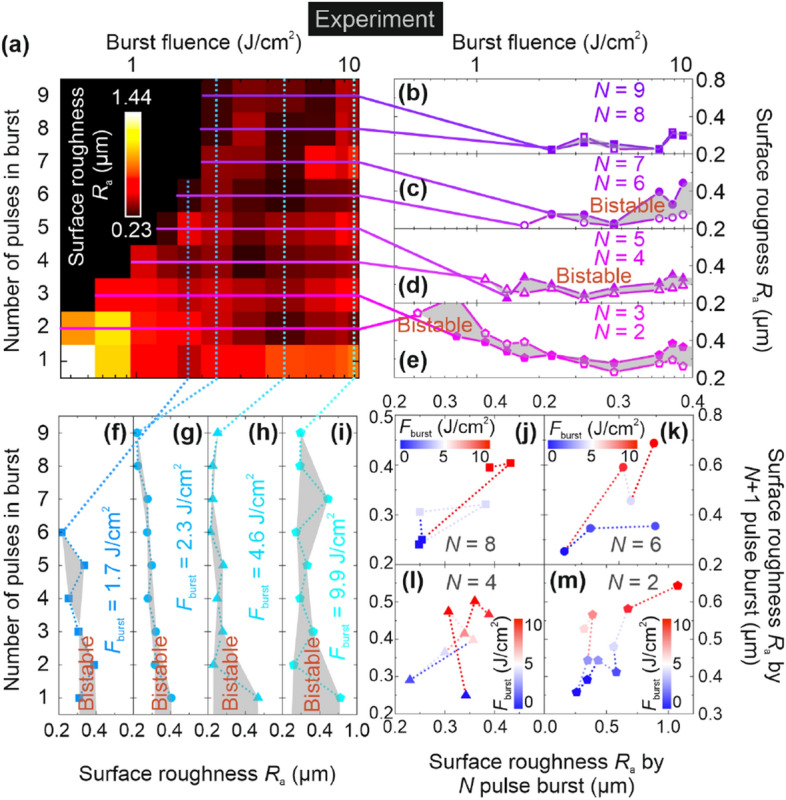
Experimental results of bi-stable behavior of surface roughness *R*_a_ of laser ablated cavity bottom. (**a**) Surface roughness *R*_a_ of laser ablated cavity bottom of copper (color scale) dependence on the burst fluence (top axis) and the number of pulses in femtosecond burst (left axis), the squares indicated by black color is not measured points because of pulse laser fluence below the ablation threshold. The surface roughness *R*_a_ (right axis) dependence on burst fluence (top axis) at different numbers of pulses in burst: (**b**) *N* = 8, 9; (**c**) *N* = 6, 7; (**d**) *N* = 4, 5; (**e**) *N* = 2, 3. Profiles of surface roughness *R*_a_ (bottom axis) dependence on the number of pulses in the burst (left axis) at different burst fluences: (**f**) 1.7 J/cm^2^; (**g**) 2.3 J/cm^2^; (**h**) 4.6 J/cm^2^; (**i**) 9.9 J/cm^2^. The surface roughness *R*_a_ created by (*N* + 1) pulse burst (right axis) dependence on surface roughness *R*_a_ created by *N* pulse burst (bottom axis) at different burst fluences (color scale): (**j**) *N* = 8; (**k**) *N* = 6; (**l**) *N* = 4; (**m**) *N* = 2. Pulse duration *τ*_FWHM_ = 210 fs, laser wavelength *λ* = 1030 nm, burst repetition rate *f*_burst_ = 100 kHz, and the temporal distance between pulses in burst *t*_pulse_ = 15.5 ns (intra-burst pulse repetition rate *f*_pulse_ = 64.7 MHz).

The surface roughness *R*_a_ of laser ablated cavity bottom dependence on laser fluence was investigated experimentally with the burst fluences ranging from 0.52 to 9.9 J/cm^2^ and 9 different numbers *N* of pulses in the burst (1, 2, 3, 4, 5, 6, 7, 8, and 9). The experiment showed bi-stability in surface roughness *R*_a_ depending on the laser processing parameters (Fig. 2).

The surface roughness *R*_a_ dependence on the burst fluence and the number of pulses in the burst are depicted in Fig. 2a. The burst fluence was increased from 0.52 to 9.9 J/cm^2^ and the number of pulses in the burst was increased from 1 to 9. The surface roughness *R*_a_ in most of the cases was slightly smaller for even numbers (*N* = 2, 4, 6, and 8) than for odd numbers (*N* = 1, 3, 5, 7, and 9) of pulses in a burst if compared to the same burst fluence at large values (2.3 J/cm^2^ − 9.9 J/cm^2^) (Fig. 2a). However, for small values of burst fluence (0.52 J/cm^2^ − 1.7 J/cm^2^) opposite behavior could be observed with a surface roughness *R*_a_ smaller for odd than for even numbers of pulses in the burst (except for *N* = 1) (Fig. 2a).

**Figure 3:**
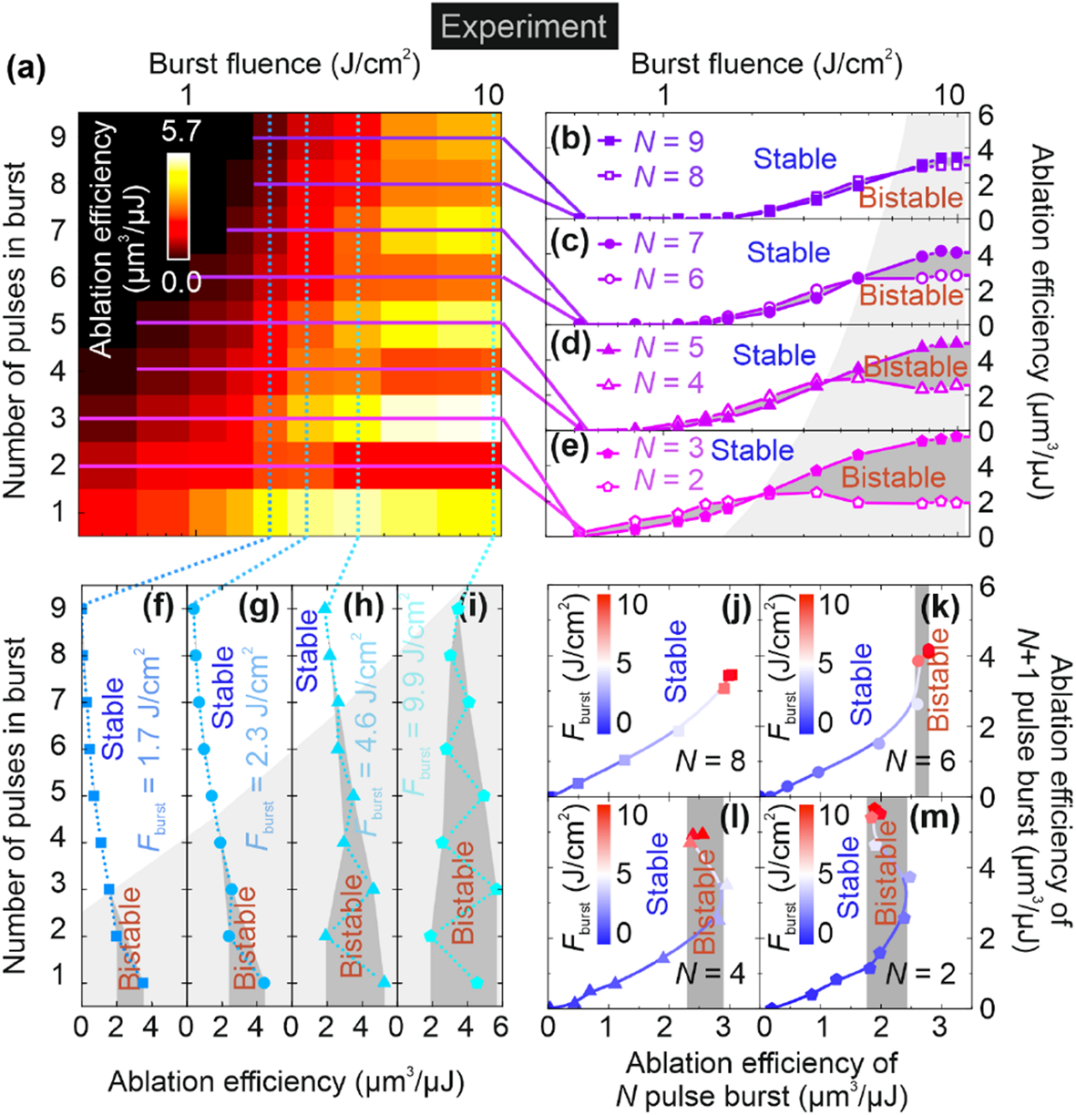
Experimental results of stable and bistable behavior of laser ablation efficiency. (**a**) Laser ablation efficiency of copper (color scale) dependence on the burst fluence (top axis) and the number of pulses in femtosecond burst (left axis), the squares indicated by black color is not measured points because of pulse laser fluence below the ablation threshold. Ablation efficiency (right axis) dependence on burst fluence (top axis) at different numbers of pulses in burst: (**b**) *N* = 8, 9; (**c**) *N* = 6, 7; (**d**) *N* = 4, 5; (**e**) *N* = 2, 3. Profiles of ablation efficiency (bottom axis) dependence on the number of pulses in the burst (left axis) at different burst fluences: (**f**) 1.7 J/cm^2^; (**g**) 2.3 J/cm^2^; (**h**) 4.6 J/cm^2^; (**i**) 9.9 J/cm^2^. Ablation efficiency of (*N* + 1) pulse in a burst (right axis) dependence on ablation efficiency of *N* pulse in a burst (bottom axis) at different burst fluences (color scale): (**j**) *N* = 8; (**k**) *N* = 6; (**l**) *N* = 4; (**m**) *N* = 2. Stable- and bi-stable laser efficiency regions are indicated by white and grey colors in (**b**–**m**). Pulse duration *τ*_FWHM_ = 210 fs, laser wavelength *λ* = 1030 nm, burst repetition rate *f*_burst_ = 100 kHz, and the temporal distance between pulses in burst *t*_pulse_ = 15.5 ns (intra-burst pulse repetition rate *f*_pulse_ = 64.7 MHz).

This bi-stability effect of surface roughness *R*_a_ depending on the burst fluence can be seen when the profiles of the color plot depicted in Fig. 2a are extracted at constant odd and even numbers of pulses in the burst (Fig. 2b–e). For example, for *N* = 8 the surface roughness *R*_a_ was slightly higher than *N* = 9 for several fluence values, and slightly lower for the rest of the fluence values with a relative difference in roughness only of 2% (Fig. 2b). For *N* = 6 surface roughness *R*_a_ was slightly lower than *N* = 7 up for all fluence values ranging from 1.7 up to 4.6 J/cm^2^ (Fig. 3c). The average relative surface roughness *R*_a_ difference increased to 39% (Fig. 2c). This ablation regime can be called the bi-stable regime (Fig. 2c). Moreover, for *N* = 4 and *N* = 5 bistable surface roughness *R*_a_ from 1.7 J/cm^2^ to 9.9 J/cm^2^ is observed (Fig. 2d). The average relative surface roughness *R*_a_ difference was 18% (Fig. 2d). A slightly different bi-stability was observed for *N* = 2 and *N* = 3 pulses (Fig. 2e). For low fluence values ranging from 0.52 to 2.3 J/cm^2^ surface roughness, *R*_a_ was higher for an even number of pulses in burst with the average relative surface roughness *R*_a_ difference of 26% (Fig. 2e). For high fluence values ranging from 3.3 to 9.9 J/cm^2^ surface roughness, *R*_a_ was higher for an odd number of pulses in burst with the average relative surface roughness *R*_a_ difference of 31% (Fig. 2e).

The bi-stability effect of surface roughness *R*_a_ depending on the number of pulses in the burst can be seen when the profiles of the color plot depicted in Fig. 2a are extracted at constant burst fluence (Fig. 2f–i). For example, for *F*_burst_ = 1.7 J/cm^2^ the surface roughness *R*_a_ sudden jumps are seen for every next number of pulses in a burst from *N* = 1 to *N* = 6 (Fig. 2f). The gradual decrease of the surface roughness *R*_a_ with an increasing number of pulses in a burst from *N* = 1 to *N* = 9 for larger values of burst fluence of *F*_burst_ = 2.3 J/cm^2^ with small sudden jumps are observed (Fig. 2g). The decrease of the surface roughness *R*_a_ with an increasing number of pulses and larger values of burst fluence of *F*_burst_ = 4.6 J/cm^2^ with large sudden jumps are observed (Fig. 2h). For the largest burst fluence of *F*_burst_ = 9.9 J/cm^2^ the bi-stable regime with sudden jumps of drastic decrease of surface roughness *R*_a_ at even numbers (*N* = 2, 4, 6, and 8) of pulses is observed (Fig. 2i). This bi-stability regime of surface roughness *R*_a_ depending on the burst fluence can be seen when the surface roughness of *N* pulse burst depending on (*N* + 1) pulse burst is extracted from color plot Fig. 2a and depicted in (Fig. 2j–m). The bi-stable regimes occur because for surface roughness *R*_a_ every even number (*N* = 2, 4, 6, and 8) several possible values of surface roughness *R*_a_ exist corresponding *N* + 1 odd numbers (3, 5, 7, and 9) every even number. The experimental results provided in Fig. 2 showed stable and bi-stable surface roughness *R*_a_ regions depending on the burst fluence and the burst length. The laser polishing regime with the lowest overall value of surface roughness *R*_a_ of 0.23 µm was found at the burst fluence of *F*_burst_ = 4.6 J/cm^2^ and the number of pulses in a burst of *N* = 6.

### Ablation efficiency

The ablation efficiencies dependence on laser fluence was investigated experimentally with the burst fluences ranging from 0.52 to 9.9 J/cm^2^ and 9 different numbers *N* of pulses in the burst (1, 2, 3, 4, 5, 6, 7, 8, and 9). The experiment showed stable and bi-stable ablation efficiency depending on the laser processing parameters (Fig. 3).

The laser ablation efficiency dependence on the burst fluence and the number of pulses in the burst are depicted in Fig. 3a. The burst fluence was increased from 0.52 to 9.9 J/cm^2^ and the number of pulses in the burst was increased from 1 to 9. The laser ablation efficiency was always smaller for even numbers (*N* = 2, 4, 6, and 8) than for odd numbers (*N* = 1, 3, 5, 7, and 9) of pulses in a burst if compared to the same burst fluence at large values (7–9.9 J/cm^2^) (Fig. 3a). However, for small values of burst fluence (0.52–2.3 J/cm^2^) opposite behavior could be observed with ablation efficiencies smaller for odd than for even numbers of pulses in the burst (except for *N* = 1) (Fig. 3a).

This stability and bi-stability effect of ablation efficiency depending on the burst fluence can be seen when the profiles of the color plot depicted in Fig. 3a are extracted at constant odd and even numbers of pulses in the burst (Fig. 3b–e). For example, for *N* = 8 the ablation efficiency was slightly higher than *N* = 9 up to fluencies of 7 J/cm^2^, and then a switch to the opposite with a relative difference in efficiency only of 13% (Fig. 3b). The ablation regime can be characterized as stable up to a bust fluence of 7 J/cm^2^ and small bifurcation starting when fluence exceeds 7 J/cm^2^ (Fig. 3b). For *N* = 6 and *N* = 7 similar behavior was observed, however, more pronounced bifurcation was observed when burst fluence increased up to 4.6 J/cm^2^ (Fig. 3c). When increasing fluence to 9.9 J/cm^2^ the relative ablation efficiency difference increased up to 37% (Fig. 3c). This ablation regime can be called the bi-stable regime (Fig. 3c). Moreover, for *N* = 4 and *N* = 5 similar stable ablation efficiency up to 4.0 J/cm^2^ is observed (Fig. 3d). However, an even more pronounced bifurcation burst fluence of 4.6 J/cm^2^ was seen with the relative ablation efficiency difference increasing up to 64% (Fig. 3d). Further increase of bi-stability was observed for *N* = 2 and *N* = 3 pulses after exceeding 2.3 J/cm^2^ the burst fluence with the relative ablation efficiency difference growing up to 101% (Fig. 3e).

The stability and bi-stability effect of ablation efficiency depending on the number of pulses in the burst can be seen when the profiles of the color plot depicted in Fig. 3a are extracted at constant burst fluence (Fig. 3f–i). For example, for *F*_burst_ = 1.7 J/cm^2^ the ablation efficiency gradually decreases for an increasing number of pulses in a burst from *N* = 1 to *N* = 9 without any sudden jumps (Fig. 3f). A similar gradual decrease of the ablation efficiency with an increasing number of pulses in a burst from *N* = 1 to *N* = 9 for a larger value of burst fluence of *F*_burst_ = 2.3 J/cm^2^ also without any sudden jumps are observed (Fig. 3g). The only exceptions and sudden jumps with a single decrease in ablation efficiency are observed for even numbers *N* = 2 (Fig. 3f,g). However, when moving to a larger burst fluence of *F*_burst_ = 4.6 J/cm^2^ the bi-stable regime with two sudden jumps of drastic decrease of ablation efficiency at even numbers (*N* = 2 and 4) of pulses is observed (Fig. 3h). For the largest burst fluence of *F*_burst_ = 9.9 J/cm^2^ the bi-stable regime with four sudden jumps of drastic decrease of ablation efficiency at even numbers (*N* = 2, 4, 6, and 8) of pulses is observed (Fig. 3i). The largest overall value of ablation efficiency of 5.66 µm^3^/µJ is observed at the burst fluence of *F*_burst_ = 9.9 J/cm^2^ and the number of pulses in a burst of *N* = 3. It exceeded by 1% the highest laser milling efficiency for copper reached in our previous work^11^. This stability and bi-stability regimes of ablation efficiency depending on the burst fluence can be seen when the ablation efficiencies of *N* pulse burst depending on (*N* + 1) pulse burst is extracted from color plot Fig. 3a and depicted in (Fig. 3j–m). The completely stable regime is observed for a large pulse value *N* = 8, with a line-type curve for all burst fluences ranging from 0.52 to 9.9 J/cm^2^ (Fig. 3j). The straight-line curve means that for one value of ablation efficiency at a burst-pulse number of *N* = 8 there is only one possible value of ablation efficiency for burst pulse number of (*N* + 1) = 9. Therefore, the regime is completely stable, and there are no sudden jumps in ablation efficiency observed. A similar stable regime is observed for the smaller pulse value of *N* = 6 with a line-type curve for burst fluences ranging from 0.52 to 4.6 J/cm^2^ (Fig. 3k). For fluences from burst fluences ranging from 4.6 to 9.9 J/cm^2^ the line goes upwards, therefore, the beginning of bi-stability is observed (Fig. 3k). The bi-stable regime occurs when the number pulse value decreases to *N* = 4, with a parabola-type curve for burst fluences ranging from 4.6 to 9.9 J/cm^2^ (Fig. 3l). The parabola-like curve is always bi-stable because for one single value of ablation efficiency at a burst-pulse number of *N* = 4 there are two possible values of ablation efficiency for a burst-pulse number of (*N* + 1) = 5. The complete bi-stability in ablation efficiency is seen in the number of pulse values decreasing to *N* = 2, with a parabola-type curve for burst fluences ranging from 2.3 to 9.9 J/cm^2^ (Fig. 3m). The experimental results provided in Fig. 3 showed stable and bi-stable ablation efficiency regions depending on the burst fluence and the burst length. Further in this work, we will provide graphical assumptions and basic principles of the shielding effect by the cloud of ablation products (particles, vapor, and plasma) in the laser ablation efficiency of parabolic dimple, which later will be included in our analytical and numerical modeling.

### Shielding versus re-deposition

The bi-stable behavior of ablation efficiency initiated by bursts of ultrafast pulses has been reported in numerous of scientific papers for copper^6,11,13,16,17^, brass^35^ and aluminum^35^. The shielding or/and re-deposition has been identified main reasons of bi-stable behavior of ablation efficiency depending in the pulse number in burst in most of the research works (Table 1).

**Table 1:** Scientific literature review of bistability in ablation efficiency depending on pulse number in burst with indicated reason of bistable behavior.

Material	Irradiation mode	Pulse duration	Wavelength (nm)	Distance between pulses	Indicated bistability reason	References
Cu	Burst	2 ps	1030	25 ns	Shielding or re-deposition	^6^
Cu	Burst, bi-burst	210 fs	1030	15.5 ns, 205 ps	Shielding or re-deposition	^11^
Cu, Kevar	Burst	1.5 ps	1064	2.3 ps	–	^13^
Cu	Burst	10 ps–210 fs	1030	15.5 ns	Shielding and redeposition	^16^
Cu	Burst	400 fs	1030	12.2 ns	Shielding and redeposition	^17^
Al	Burst	350 fs	1035	17 ns	Shielding or re-deposition	^35^
Brass	Burst	350 fs	1035	25 ns	Shielding or re-deposition	^35^

Shielding together with the redeposition effect are wildly attributed to bistable ablation efficiency in the field, however, the mostly it is only hypothesis with no direct observation of the phenomenon. The shielding and re-deposition of copper target has been experimentally studied by shadowgraph technique with similar experimental conditions (wavelength 1030 nm, pulse duration 400 fs, repetition rate 400 kHz, delay between pulses 12.2 ns, fluence 0.69 J/cm^2^, spot diameter 44 µm)^17^. The shielding effect by ablation cloud has been directly visualized via the shadowgraph technique, however, no clear experimental evidence of re-deposited material was seen^17^. Generally, for the re-deposition, the negative ablation depth for the second sub-pulse in burst is supposed to be measured, resulting the efficiency decrease for two sub-pulse burst below 50%, if compared with a single pulse ablation efficiency of 100%. Since some material ejected by the first pulse is reposited by the second, and the total volume ablated by both pulses is less than that ablated by a single first pulse is divided by double pulse energy of two sub-pulses. Even though, we see such efficiency drop down to 38% in our experiment: at single pulse ablation with peak pulse fluence of 4.6 J/cm^2^ for most efficient ablation of 5.3 µm^3^/µJ to efficiency drop of 2.0 µm^3^/µJ for double pulse ablation with peak pulse fluence of 4.4 J/cm^2^. We believe that this can be assumed as experimental evidence of re-deposition in our experimental conditions. However, for the simplicity of our toy model we neglected re-deposition and concentrated to the shielding by ablation cloud.

### Toy model

**Figure 4:**
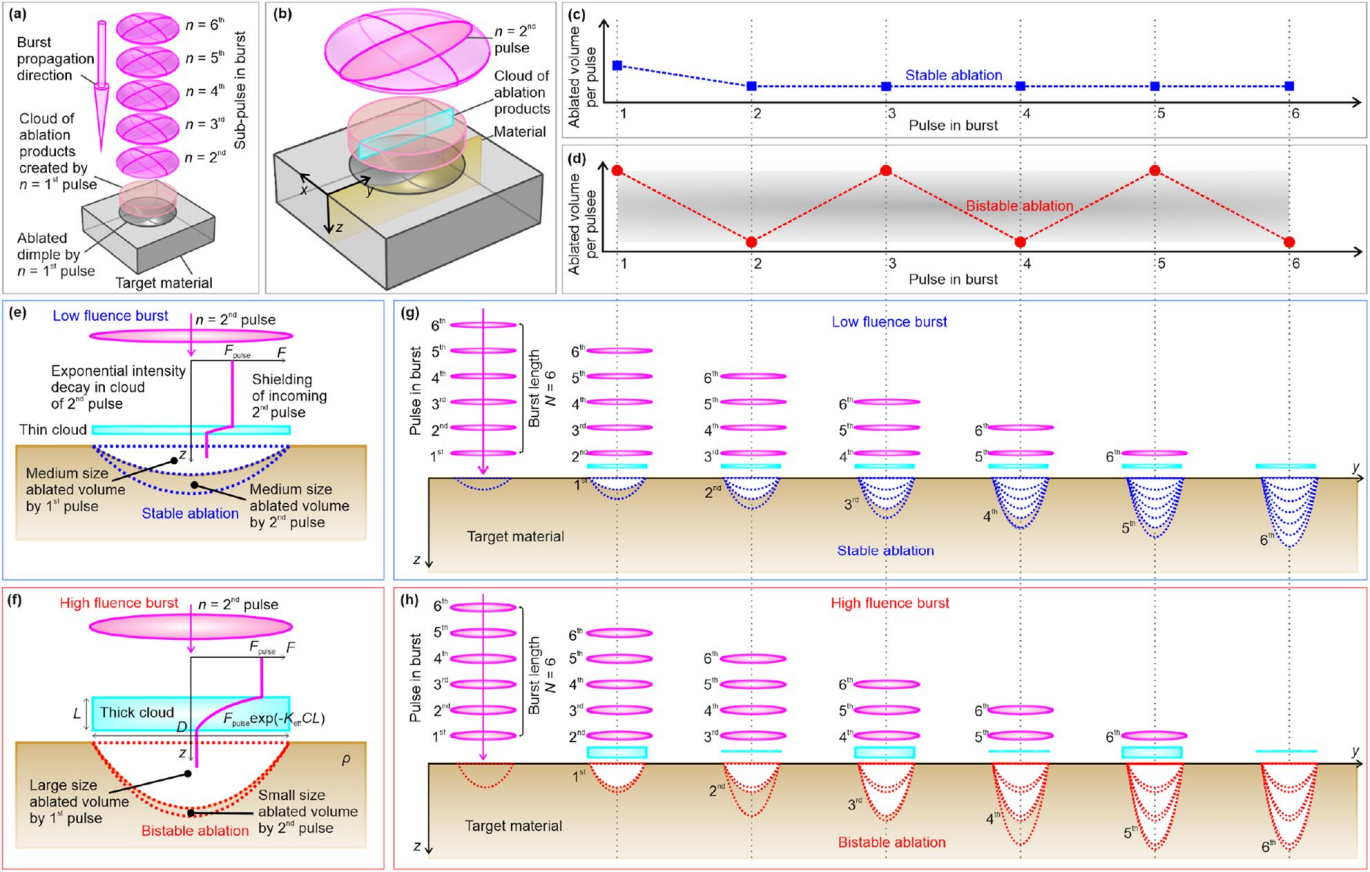
Graphical representation of toy model of the laser ablation with shielding effect and stable or bi-stable ablation. (**a**) 3D representation of target material irradiated with laser burst with the indicated target material, parabolic dimple ablated by 1st pulse in the burst, the cloud of ablation debris created by the first pulse in position above the dimple with cylindrical shape indicated by pink transparent color, the burst with five remaining (2nd, 3rd, 4th, 5th, and 6th) pulses indicated by magenta ellipsoids, and the burst propagation direction indicated by the pink arrow. (**b**) 2D cross-section on the 3D representation of target material with parabolic dimple ablated by the first pulse in the burst, cloud of ablation debris created by the first pulse, and the second pulse in a burst. Schematic representation of ablated volume per pulse dependence on the pulse in burst: (**c**) stabile ablation (blue color—low burst fluence), (**d**) bi-stable ablation (red color—high burst fluence). The second pulse in the burst is partially shielded by the debris cloud, therefore, with exponential intensity drop defined by the Beer-Lambert-Bouguer absorption law. The volume ablated by the second pulse is always smaller than the volume ablated by the first pulse in the burst. For the low burst fluence ablation is stable with a similar ablated volume per pulse (**c**), however, for high burst fluence ablation is bistable with a large ablated volume per pulse for an even number, and small for the odd number of pulses per burst (**d**). 2D cross-section of geometry with indicated parabolic dimple ablated by the second pulse and the cloud of ablation products (particles, vapor, and plasma) above the dimple is indicated by cyan rectangle: (**e**) stabile ablation (low burst fluence), (**f**) bistable ablation (high burst fluence). The sequence of ablation using burst with the burst length of *N* = 6 pulses: (**g**) stabile ablation, (**h**) bi-stable ablation. Dashed lines where blue (stable ablation) and red (bi-stable ablation) lines indicate the volume ablated by each of the pulses in a burst in (**e**–**h**).

The two-dimensional (2D) and three-dimensional (3D) graphical representation of the new toy model for laser ablation of parabolic dimples including the shielding effect of every next pulse by the cloud of ablation products created by every previous pulse is presented in Fig. 4.

Principal 3D scheme of the target material irradiated by a laser burst with the specified target material, the parabolical shape dimple ablated by the 1st pulse of the burst, the ablation debris cylindrical cloud resulting from the 1st pulse above the ablated crater, indicated by pink transparent color, the burst with the remaining five (2nd, 3rd, 4th, 5th, and 6th) pulses indicated by purple ellipsoids, the direction of propagation of the laser burst indicated by a pink arrow, and indicated *xyz* Cartesian coordinate system is depicted Fig. 4a. 2D cross-sections on a 3D image of the target material with a parabolic dimple ablated by the first pulse in the burst, the ablation debris cloud resulting from the first pulse, and the second pulse in the burst, are represented in Fig. 4b. A principal behavior of the ablated volume per pulse on pulse sequence number in burst for stable and bi-stable ablation scenarios is provided in Fig. 4c,d, respectively. The 2nd pulse is partially shielded by the debris cloud created by 1st pulse, so its intensity decreases exponentially according to the Beer-Lambert-Bouguer absorption law, depending on cloud thickness and the concentration of particles, vapor, and plasma in the cloud. The ablated volume of the 2nd pulse is always smaller than the ablated volume of the first pulse because of shielding. At low burst fluence, the ablation is stable and the ablation volume per pulse is similar in size for all pulse numbers except (*n* = 1) (Fig. 4c), but at high burst fluence the ablation is bi-stable and at even number of pulses the ablation volume per pulse is larger than for the odd pulse number (Fig. 4d). The graphical representation of a cross-section of the 2D geometry with the parabolic cavity ablated by the second pulse, and the cloud of ablation products (particles, vapors, and plasma) above the cavity is indicated by the cyan rectangle for a stable ablation process at low burst fluence provided in Fig. 4e, and for bi-stable ablation process at high burst fluence in Fig. 4f. At low fluence the medium size ablated volume is removed from the material by the first pulse which creates a thin cloud at low concentration, therefore, a weak shielding effect is observed. It results in a minor change in laser fluence at the target material for the second pulse, and it causes almost the same ablated volume per pulse. The process continues for every next pulse in burst and stable ablation with constant ablated volume per pulse Fig. 4e. At high fluence the large size ablated volume is removed from the material by the first pulse which creates a thick cloud at high concentration, therefore, a strong shielding effect is observed. It results in a major change in laser fluence at the target material for the second pulse, and it causes a big decrease in ablated volume per pulse for the second pulse. The process continues for every next pulse in burst and bi-stable ablation with jumping ablated volume per pulse observed Fig. 4f. The graphical representation of the sequence of the ablation process for the burst with the length of *N* = 6 pulses for the stable ablation with volume per pulse constant for every pulse is given in Fig. 4g and for the bi-stable ablation with jumping volume per pulse is given in Fig. 4h. The dashed lines indicate the volume ablated with each of the pulses during the burst irradiation. The amount of ablation products is directly related to the volume ablated by the first pulse, thus, the volume ablated by the second pulse depends on the volume ablated by the first pulse. A similar relationship can be extended for every next pulse (third, fourth, fifth, sixth, etc.). Furthermore, the generalized rule for the (*n* + 1)th pulse ablation efficiency and its dependence on the ablation efficiency of the previous *n*th pulse, because partial shielding by the cloud created by *n*th pulse, can be created. Further in the work, we will provide detailed analytical and numerical modeling of the shielding effect by ablation products (particles, vapor, and plasma) which was included in toy model of the laser ablation efficiency of parabolic dimple for the first time.

In our ablation model, the partial shielding of the incoming pulse by the cloud of ablation products created by the previous pulse in the burst is taken into account. The dimple with the shape of a paraboloid of revolution is created by ablation with the first pulse in the burst. The cloud of ablation debris is created above the dimple ablated by the first pulse. The second pulse in the burst is partially shielded by the debris cloud, as a consequence, the pulse with the smaller intensity reaches the target material, therefore, the volume ablated by the second pulse is affected by the volume ablated by the first pulse. If the large volume is ablated by the first pulse, then a big cloud of ablation debris is created, and shielding of the next pulse greatly influences the amount of radiation that reaches the sample. Therefore, the ablated volume of the second pulse is very low. It also creates a very small amount of ablation debris. Thus, the third pulse is not shielded by the cloud and the volume ablated by the third pulse is large again. And the bistable ablation rate with a large ablated volume for the odd numbers of pulses (first, third, fifth, etc.) and a small ablated volume of even numbers of pulses (second, fourth, sixth, etc.) continues. In general, the ablation efficiency of (*n* + 1)th pulse is influenced by the ablation efficiency of the *n*th pulse, because the intensity of (*n* + 1)th is partially shielded by the cloud created by *n*th pulse, and the part of absorption is proportional to the ablation efficiency of the *n*th pulse. The ablated volume *V*_*n*_ per pulse in the burst can be expressed by^32–34^:

2 $$V_{n} = \frac{{\pi w^{2} \delta }}{4}\ln \left( {\frac{{F_{n} }}{{F_{n}^{{{\text{th}}}} }}} \right)^{2} ,$$

where *n* is the integer number indicating the position of the pulse in the burst, *w* is the Gaussian beam radius on the surface of the sample*, δ* is effective penetration depth, *F*_*n*_ is peak laser fluence in the center of the beam of *n* pulse, *F*^th^_*n*_ is the ablation threshold, which depends on the number (*n* − 1) of previous pulses which has already affected the material. The surface of the target material is modified after each successive pulse. For example, after the first pulse, it is expected that for the second pulse, the target surface is no longer flat, and is expected to have a parabolically shaped surface, with certain roughness. However, mathematically there is no equation describing ablated volume by Gaussian beam depending on fluence on the parabolically shaped surface with a certain roughness, thus we use approximation with the same formula (2) for the multi-pulse regime, neglecting surface modifications by previous pulses. A similar approach has been used in pioneering research works related to fluence optimization for max efficiency, where the volume of all succeeding pulses in a multi-pulse regime has been calculated by the same equations, nevertheless, the model had good coincidence experiment^32–34^.

In our proposed ablation model, we assume, that, the incoming laser fluence of the *n*th pulse is attenuated by a cloud of particles, vapor, and plasma created from ablation products of (*n* − 1) pulse in a burst. We further show the derivations of the mathematical expression of the ablation efficiency recurrence relation. The mass *m*_*N*_ of ablation products created by *n*th pulse can be expressed:

3 $$m_{n} = \rho V_{n} = C_{n} L_{n} \frac{{{\uppi }D_{n}^{2} }}{4},$$

where *ρ* is the abated material density, *V*_*n*_ is the volume ablated by *n*th pulse described by Eq. (2), *C*_*n*_ is the total concentration of shielding cloud (particles, vapor, and plasma), *L*_*n*_ is the averaged cloud thickness, and *D*_*n*_ cloud diameter (see Fig. 4f). It is assumed in our model, that that cloud diameter is approximately equal to the ablated dimple diameter by *n*th pulse:

4 $$D_{n}^{2} = 2w^{2} \ln \left( {\frac{{F_{n} }}{{F_{n}^{{{\text{th}}}} }}} \right).$$

**Figure 5:**
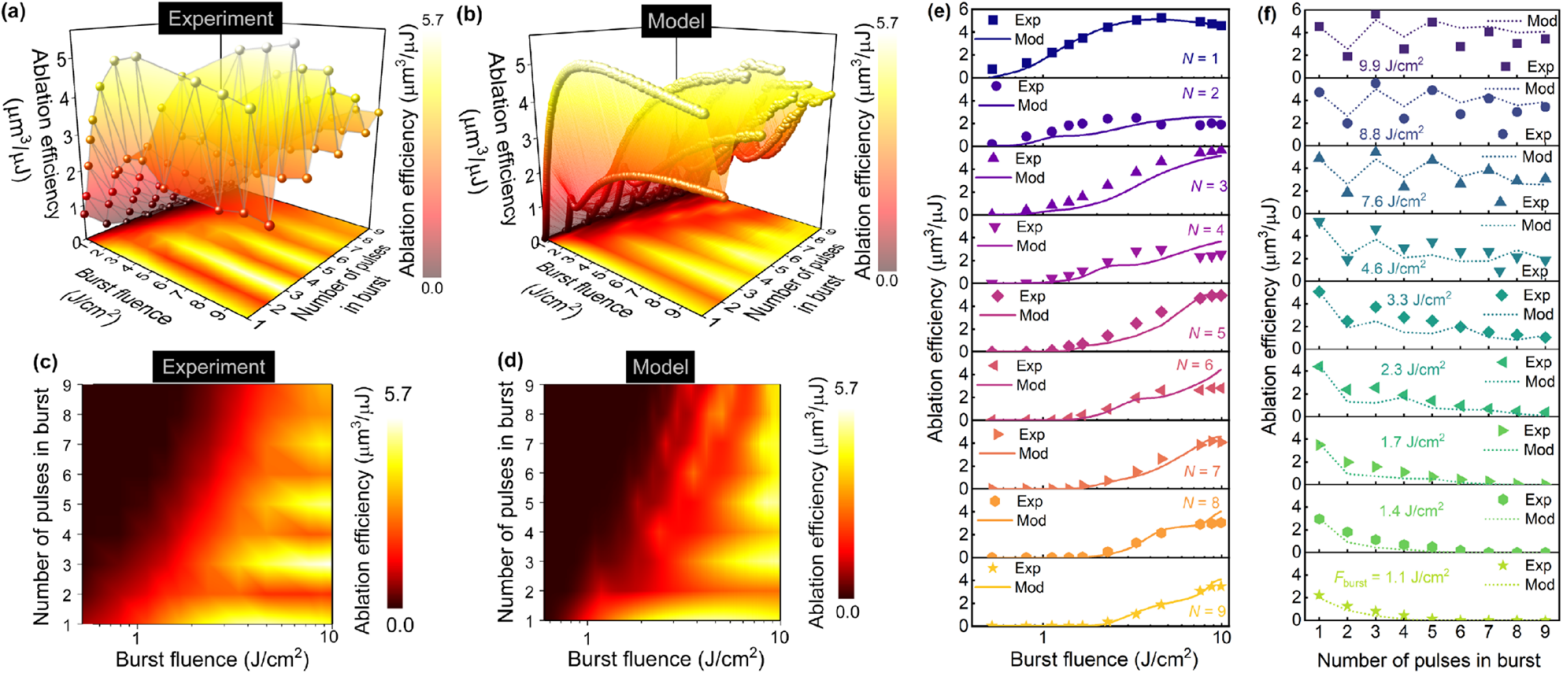
Comparison of the experimental data to the results of theoretical toy modeling. Laser ablation efficiency of copper (color scale) dependence on the burst fluence and the number of pulses in femtosecond burst: (**a**, **b**) 3D views and (**c**, **d**) 2D maps of experimental and modeling results. (**e**) 1D profiles of ablation efficiency dependence on burst fluence at different numbers of pulses in burst ranging from *N* = 1 (top) to *N* = 9 (bottom). (**f**) 1D profiles of ablation efficiency dependence on the number of pulses in a burst at different burst fluences ranging from *F*_burst_ = 1.7 J/cm^2^ (bottom) to 9.9 J/cm^2^ (top). Experimental data points (dots) are quantitatively compared with the predictions of our theoretical model (lines) in (**e**, **f**). Laser parameters: pulse duration *τ*_FWHM_ = 210 fs, laser wavelength *λ* = 1030 nm, burst repetition rate *f*_burst_ = 100 kHz, the temporal distance between pulses in burst *t*_pulse_ = 15.5 ns (intra-burst pulse repetition rate *f*_pulse_ = 64.7 MHz).

It is also considered, that the cloud of ablation products expands fast in longitudinal beam propagation *z*-direction. The lateral expansion of plume at 15 ns time scale might be significant, however, the recent research work shows the plasma expansion induced by femtosecond laser with a wavelength of 800 nm pulse duration of 50 fs is in the longitudinal direction rather than in the transverse one up to 100 ns^36^. The semi-spherical shock wave is usually recorded with expansion in both longitudinal and transverse directions, however, plasma plume expansion is mostly exposed in the longitudinal direction. The mechanisms of ultrafast GHz burst fs laser ablation of copper have been recently investigated by time-resolved scattering imaging, emission imaging, and emission spectroscopy in^37^. Similar conditions to our experimental setup have been used utilizing pulses with 500 fs duration at 1030 nm and GHz fs bursts (50 and 200 pulses at 1.28 GHz repetition rate) with focused beam diameter of 16 µm. After single-pulse irradiation, Cu plasmas were observed for approximately 30 ns. Also, Cu nanoparticle ejections with speeds ranging from 100 m/s to 350 m/s have been recorded, depending on particle type. At such speeds, the copper particles travel from 1.6 to 5.4 µm distances during sub-pulse delay of 15.5 ns, and from 12.4 to 43.4 µm, during 9 sub-pulse burst duration of 124 ns, which are comparable to spot radiuses ranging from 21 to 95 µm used in our experiments. Thus, it can be considered that the transverse expansion of the plasma plume can be neglected with some tolerance, as cloud expansion is much slower in transverse *x-* and *y*-directions since ejected particles have the velocity vectors mostly in the *z*-direction^38–40^. Therefore, for the short time interval, the shielding cloud concentration multiplied by the average cloud thickness will be constant and equal to ablated mass divided by dimple area as *C*_*n*_*L*_*n*_ = 4* m*_*n*_/(π*D*_*n*_^2^) = const., because the diameter squared of ablated area, and ablated mass remains constant. It is assumed in our ablation model that, the incoming laser radiation is attenuated by the shielding effect which results in a decrease of incident fluence which reaches the material and results in a decrease in laser ablation efficiency. Thus, the attenuated fluence that reaches the surface of target materials after passing through a cloud of ablation products can be expressed by Beer-Lambert-Bouguer absorption law^41,42^:

5 $$F_{n + 1} = F_{{{\text{pulse}}}} \exp \left( { - K_{{{\text{eff}}}} C_{n} L_{n} } \right),$$

where *K*_eff_—effective shielding coefficient which takes into account attenuation by particles, vapor, and plasma generated by the previous pulse. By analytically solving Eqs. (1), (2), (3), (4) and (5) we get the final equation of the ablation efficiency of (*n* + 1)th pulse, which is influenced by ablation efficiency by the previous *n*th pulse:

6 $$\eta_{n + 1}^{{{\text{pulse}}}} = \frac{\delta }{{2F_{{{\text{pulse}}}} }}\left( {\ln \left( {\frac{{F_{{{\text{pulse}}}} }}{{F_{n + 1}^{{{\text{th}}}} }}} \right) - K_{{{\text{eff}}}} \rho \sqrt {\frac{{\delta F_{{{\text{pulse}}}} \eta_{n}^{{{\text{pulse}}}} }}{2}} } \right)^{2} .$$

The detailed derivation of Eq. (6) is provided in the Methods section. This type of equation is known as recurrence relation because the efficiency *η*^pulse^_*n*+1_ of (*n* + 1)th pulse is a function of efficiency *η*^pulse^_*n*_ of *n*th pulse as *η*^pulse^_*n*+1_ = *f*(*η*^pulse^_*n*_), mathematically defined by Eq. (6). Bi-stability, as well as sudden jumps, occur in ablation efficiency depending on the number of pulses in bursts with the response to changes of the control parameter peak laser fluence in the pulse *F*_pulse_. Further research has been conducted in this work, to compare theoretical modeling using Eq. (6) results with experimental data provided in Fig. 3.

The ablation threshold of (*n* + 1)th pulse dependence on the number of pulses *n* applied before can be expressed as^43,44^:

7 $$F_{n + 1}^{{{\text{th}}}} = F_{\infty }^{{{\text{th}}}} + \left( {F_{1}^{{{\text{th}}}} - F_{\infty }^{{{\text{th}}}} } \right){\text{e}}^{ - kn} ,$$

where *F*^th^_1_ is the single-shot threshold, the *F*^th^_∞_ multi-shot threshold, *k* is the empirical parameter in the exponent which characterizes the strength of incubation leading to an early reduction of the threshold. The ablation efficiency dependence on the peak laser fluence *F*_pulse_ in the pulse for the first pulse in the burst (single pulse irradiation regime) can be evaluated by using classical Eq. (1). The experiment results have good agreement with the numerical calculation results of ablation efficiency for single pulse irradiation (see Fig. 1e). By having calculated the ablation efficiency for the single pulse *η*^pulse^_1_ it is easy to calculate the ablation efficiency for the second *η*^pulse^_2_ using Eq. (6). Then, knowing the ablation efficiencies *η*^pulse^_2_ for the second pulse, the efficiency *η*^pulse^_3_ for the third pulse in the burst using can be easily calculated in same Eq. (6). Similarly, having *η*^pulse^_3_ one can easily calculate *η*^pulse^_4_, and having *η*^pulse^_4_ easy to calculate *η*^pulse^_5_, etc. Later, the ablation efficiency of the burst *η*^burst^_*N*_ can be evaluated as the average value of ablation efficiencies of all pulses in the burst *η*^pulse^_*n*_:

8 $$\eta_{N}^{{{\text{burst}}}} = \frac{1}{N}\sum\limits_{n = 1}^{N} {\eta_{n}^{{{\text{pulse}}}} } ,$$

where *N* is the number of the pulses in the burst (burst length), *n* is the number indicating the position of the pulse in the burst*.* The burst fluence *F*_burst_ can be computed by multiplying a single pulse fluence by the burst length as *F*_burst_ = *NF*_pulse_. The single pulse fluence *F*_pulse_ was recalculated to the burst fluence in the numerical computation and the experiment.

### Simulation versus experiment

The laser ablation efficiency for copper has been tested experimentally and theoretically at burst different fluences and burst lengths. The ultrafast laser with duration *τ*_FWHM_ = 210 fs, laser wavelength *λ* = 1030 nm, burst repetition rate *f*_burst_ = 100 kHz, the temporal distance between pulses in burst *t*_pulse_ = 15.5 ns (intra-burst pulse repetition rate *f*_pulse_ = 64.7 MHz) was used to ablate an array of rectangularly shaped cavities in the copper plate, and ablation efficiency was measured (Fig. 5a,c).

Equations (6), (7), and (8) have been numerically solved by using symbolic and numeric computing software (Maple, Maplesoft). The parameters values used in numerical simulation of toy model equations together, with parameter values obtained in this work as fit of experimental data points together with closest literature values reported for copper using similar laser is provided in Table 2.

**Table 2:** Physical parameter values used in toy model simulations together with values retrieved in this work as fit parameters of experimental data points and its comparison to closest literature values obtained for copper using similar laser.

Physical parameter	Used in toy model simulation	Achieved in this work as fit of experimental data	Closest literature value	Literature laser parameters	References
*F*^th^_1_ (J/cm^2^)	0.59	0.59 ± 0.02	0.51 ± 0.08	1030 nm, 280 fs, 60 kHz	^45^
*δ* (nm)	113	113 ± 5	115	1030 nm, 10 ps, 1 kHz	^46^
*F*^th^_∞_ (J/cm^2^)	0.21	–	0.211	800 nm, 250 fs, 1 kHz	^47^
*k*	0.9	0.84 ± 0.14	*F*^th^_2_ = 0.4 J/cm^2^ *F*^th^_3_ = 0.25 J/cm^2^ *F*^th^_20_ = 0.2 J/cm^2^	1030 nm, 500 fs, 100 kHz, 25 ns	^21^
*ρ* (g/cm^3^)	8.96	–	8.96	–	^48^
*K*_eff_ (m^2^/g)	2.5	–	*µ*/*ρ*_m_ = 8.8 m^2^/g	1050 nm	^37^

The fit parameters *F*^th^_1_ = 0.59 ± 0.02 J/cm^2^, *δ* = 113 ± 5 nm were achieved as a fit of experimental data by Eq. (1) in Fig. 1e. This is a standard procedure for obtaining two physical parameter values to define a single pulse ablation threshold and effective penetration depth. The obtained threshold value coincides well with the single pulse threshold literature value *F*^th^_1_ = 0.51 ± 0.08 J/cm^2^ obtained with a similar laser (1030 nm, 280 fs, 60 kHz)^45^. It also corresponds well to literature values of copper oxide removal threshold of *F*^th^_1_ = 0.57 ± 0.03 J/cm^2^ (1064 nm, 10 ps, 100 kHz)^49^ and *F*^th^_1_ = 0.62 J/cm^2^ (1064 nm, 12 ps, 100 kHz)^50^. However, slightly higher copper ablation threshold values were obtained with femtosecond lasers at shorter wavelength *F*^th^_1_ = 0.87 J/cm^2^ (800 nm, 130 fs, 1 kHz)^51^ and *F*^th^_1_ = 0.86 J/cm^2^ (800 nm, 250 fs, 1 kHz)^47^. A similar single pulse penetration depts of *δ* = 115 nm has been declared in literature for a similar for laser (1030 nm, 10 ps, 1 kHz)^46^. Those parameters (*F*^th^_1_ = 0.59 J/cm^2^, *δ* = 113 nm) were used in further numerical simulations of toy model equations. The value of multi-shot ablation threshold of *F*^th^_∞_ = 0.21 J/cm^2^ was used in toy modeling and was taken from literature as closest multi-pulse threshold value of *F*^th^_1000_ = 0.211 J/cm^2^ experimentally retrieved using similar femtosecond laser (800 nm, 250 fs, 1 kHz)^47^. It coincides well with other literature multi-pulse threshold values of *F*^th^_1000_ = 0.19 J/cm^2^ (1064 nm, 10 ps, 100 kHz)^25^ and *F*^th^_∞_ = 0.18 J/cm^2^ (800 nm, 130 fs, 1 kHz)^51^. The incubation strength of *k* = 0.9 was used in numerical calculations of toy model. The similar value of *k* = 0.84 ± 0.14 was retrieved as fit parameter by Eq. (7) utilizing copper ablation threshold values with highest decay (*F*^th^_2_ = 0.4 J/cm^2^, *F*^th^_3_ = 0.25 J/cm^2^, and *F*^th^_20_ = 0.2 J/cm^2^) experimentally obtained by femtosecond 40 MHz burst laser (1030 nm, 500 fs, 100 kHz, 25 ns) in^21^. Similar values of *k* = 0.9 ± 0.7 (10 kHz) and *k* = 0.6 ± 0.3 (1 MHz) were retrieved as fit parameter by Eq. (7) utilizing experimental multi-pulse copper threshold values obtained using green picosecond 82 MHz burst laser (532 nm, 10 ps, 10 kHz–1 MHz, 12.2 ns) in^52^. In all our fits of copper threshold literature values by Eq. (7) the constant single pulse and multi-shot threshold values *F*^th^_1_ = 0.59 J/cm^2^ and *F*^th^_∞_ = 0.21 J/cm^2^ were used. The density of solid copper of *ρ* = 8.96 g/cm^3^ was used in numerical calculations^48^.

The only one physical constant used a free parameter in our toy model simulations was the attenuation coefficient of ablation cloud *K*_eff_, because its value could not be found in the scientific literature. Nevertheless, we used a value which is comparable to mass attenuation coefficient (also known as the mass absorption coefficient) of solid or liquid copper. The absorption coefficient of solid copper can be evaluated by Eq. (18) utilizing, the extinction coefficient of *κ* = 6.59 for copper at *λ* = 1050 nm wavelength reported in^53^, which gives the absorption coefficient of *µ* = 7.9 × 10^5^ 1/cm. The mass attenuation coefficient can be evaluated by Eq. (19), having solid copper mass density of *ρ*_m_ = *ρ* = 8.96 g/cm^3^, one can evaluate the mass absorption coefficient of *µ*/*ρ*_m_ = 8.8 m^2^/g. The ablation cloud mostly consists of copper vapor, copper nanoparticles and liquid copper, therefore, evaluation the mass attenuation coefficient for liquid copper would make sense. The density of molten copper of *ρ*_m_ = 7.90 g/cm^3^ and extinctions efficient *κ* = 2.94 at *λ* = 532 nm wavelength can be taken from^37^. It gives the absorption coefficient of *µ* = 7.0 × 10^5^ 1/cm and mass attenuation coefficient of *µ*/*ρ*_m_ = 8.9 m^2^/g for liquid copper. However, for example the extinction coefficient and absorptivity of nanoparticles might be several times lower than the bulk which also depends on the wavelength^54^. Taking it to account that ablation cloud consists of plasma, vapor, nanoparticles and liquid copper, causing internal reflections and light scattering within the cloud, the reduction of attenuation should be considered. We have chosen approximately three the half times lower attenuation coefficient of *K*_eff_ = 2.5 m^2^/g than evaluated for liquid or bulk copper for our toy model simulations of the ablation cloud which gave the best coincidence of numerical to the experimental results. In real experimental conditions, the absorption coefficient of plasma is not constant, because it depends on density and temperature of ablation cloud, and it should drastically differ for odd and even pulses. The similar behavior should also apply to effective penetration depth. However, we wanted to keep our toy model as simple as possible, with all coefficients as constant values.

The calculated ablation efficiency versus burst fluence and burst length is given in Fig. 5b,d, which is in good agreement with experimental data provided in Fig. 5a,c. Furthermore, the data of experimentally measured ablation efficiencies were quantitatively compared to the numerical computation results of the model equations in Fig. 5e,f. The series of cross-sections from the experiment data and modeling results with burst lengths ranging from *N* = 1 to *N* = 9 is shown as a function of burst fluence (Fig. 5e). Our simulation results at each value of the number of pulses in bursts were in good agreement with the ablation efficiency measurements. Also, the series of cross-sections from the experiment data and modeling results with burst fluences ranging from *F*_burst_ = 1.7 J/cm^2^ to 9.9 J/cm^2^ are shown as a function of burst length (Fig. 5f). The results of numerical computation of our new toy model equations at each value of the burst fluence were also in good coincidence with experimental values of the ablation efficiency.

### 3D cavity milling

**Figure 6:**
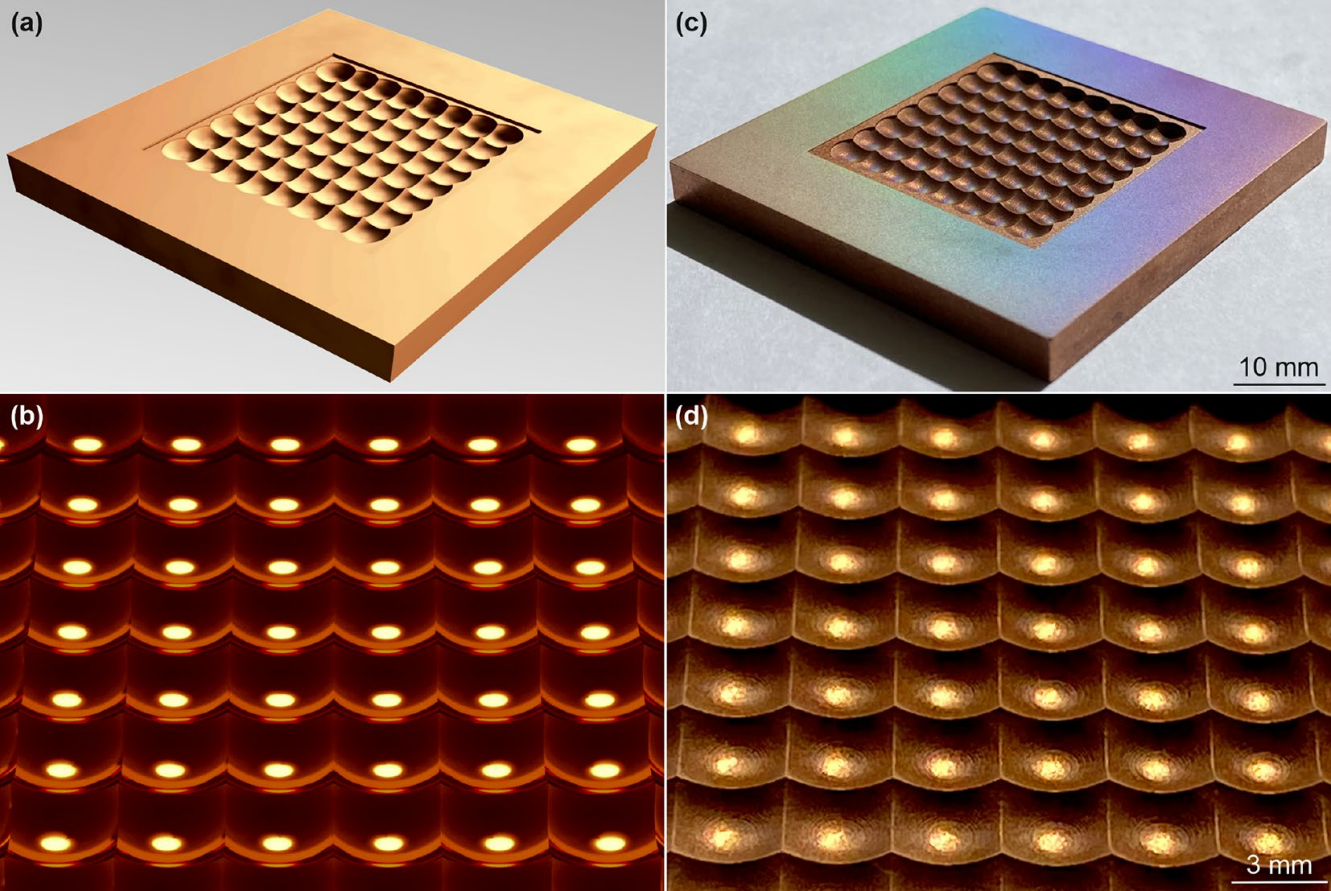
Examples of efficient laser layer-by-layer milling of 3D cavity in copper plate: (**a**) CAD model rectangular array of overlapping hemispheres (compound eye); (**b**) rendered CAD visualization perfectly smooth array of overlapping hemispheres; (**c**) digital camera image of laser milled part with an array of overlapping hemispheres; (**d**) image of the light refraction by laser milled array of overlapping hemispheres. Laser parameters: *N* = 3 pulses per burst, pulse duration *τ*_FWHM_ = 210 fs, laser wavelength *λ* = 1030 nm, burst repetition rate *f*_burst_ = 100 kHz, the temporal distance between pulses in burst *t*_pulse_ = 15.5 ns (intra-burst pulse repetition rate *f*_pulse_ = 64.7 MHz), the lateral distance between bi-directional beam scanning lines of *h* = 10 µm, beam scanning speed of *v*_*x*_ = 0.33 m/s, laser power output *P* = 7.3 W, bust fluence *F*_burst_ = 9.9 J/cm^2^, ablation efficiency *η* = 5.66 µm^3^/µJ, material removal rate d*V*/d*t* = 2.48 mm^3^/min, copper plate dimensions 50 × 50 × 5 mm^3^, laser milled cavity transverse dimensions 30 × 30 mm^2^, max milled depth 2.0 mm.

The burst mode laser micro-machining quality was evaluated by milling a complicated periodical 3D cavity, utilizing a layer-by-layer removal method^16^, starting at the sample’s top, and ending at the cavity bottom, where the micro-machining was accomplished (Fig. 6).

The 3D computer-aided design model of the cavity with a rectangular array of overlapping hemispheres, similar to compound eyes^55,56^ was created (Fig. 6a). The rendered CAD visualization reflected light by an array of overlapping hemispheres with perfect smoothness is depicted in Fig. 6b. The CAD model was sliced into parallel layers with constant thickness by using special software (DMC PRO, Direct Machining Control). After each of the layers was removed, the distance between the focusing lens and the sample surface was modified to keep the beam diameter and its related optimal laser fluence on the ablated surface. The burst fluence was set at 9.9 J/cm^2^, and the 3 pulses per burst regime was chosen as processing parameters for the most efficient ablation using burst mode. Complex periodical 3D cavities of overlapping hemispheres were laser machined with a material removal rate of 2.48 mm^3^/min (Fig. 6c). The high-quality cavity bottom was smooth with a surface roughness *R*_a_ of 0.4 µm. The specimen was further polished with burst of fluence of *F*_burst_ = 4.6 J/cm^2^ and the number of pulses in a burst of *N* = 6 and surface roughness *R*_a_ of 0.23 µm was reached at plain areas of the copper specimen. The rainbow coloring is seen because of the laser-induced periodical surface structures (LIPSS)^55,57,58^ or oxidation and nanostructures^59^ created by the laser polishing procedure^60^ (Fig. 6c). The sunlight reflected light by laser milled array of overlapping hemispheres is depicted in Fig. 6d.

## Conclusions

To conclude, the shielding effect was included in the toy model of laser ablation efficiency using MHz burst irradiation for the first time in this work. The incoming laser radiation attenuation by plasma, vapor, and particle cloud which was created from ablation products of the previous pulse was incorporated by modeling equations of ablation efficiency. The numerical results of model equations showed stable and bi-stable ablation efficiency depending on the burst fluence and burst length which coincided well with the experimental data. The ablation was stable for small values of burst fluence and/or large values of burst length because the small amount of ejected material by the previous pulse caused a weak shielding effect for the next pulse and resulted in small or almost no variation in ablation volume per pulse. However, at the high burst fluence values and/or low burst lengths, the high amount of ejected material by the previous pulse caused a strong shielding effect and a drastic decrease in ablated volume per pulse for the next pulse for every even number of femtosecond pulse in the MHz burst. The stability and bi-stability in ablation efficiency were controlled by varying the laser fluence and the number of pulses in the burst. The maximal possible laser milling efficiency with the value of 5.66 µm^3^/µJ which exceeds the highest value recorded in scientific literature has been achieved in our work by choosing optimal burst fluence together with burst length. Complex 3D cavities of high quality were laser machined and further polished by using burst processing regimes with the highest ablation rate and lowest roughness.

## Methods

### Detailed description of the toy model

Here we derive the recurrence relation of ablation efficiency leading to bi-stability. The ablation crater created by Gaussian beam pulse has parabolic shape, with characteristic volume, depending on beam radius, effective penetration depth, peak fluence to threshold ratio, which was mathematically derived and justified in research works^32–34^. Depth of laser ablated parabolic dimple ablated by *n*th pulse in the burst:

9 $$H_{n} = \delta \ln \left( {\frac{{F_{n} }}{{F_{n}^{{{\text{th}}}} }}} \right),$$

where *δ* is effective penetration depth, *F*_*n*_ is peak laser fluence in the center of the beam of *n* pulse, *F*^th^_*n*_ is the ablation threshold, depending on the number of pulses applied. Diameter of laser ablated parabolic dimple ablated by *n*th pulse in the burst:

10 $$D_{n}^{2} = 2w^{2} \ln \left( {\frac{{F_{n} }}{{F_{n}^{{{\text{th}}}} }}} \right),$$

where *w* is the Gaussian beam spot radius on the sample surface. The volume of the dimple with the shape of a paraboloid of revolution:

11 $$V_{n} = \frac{\pi }{8}H_{n} D_{n}^{2} .$$

The volume of laser ablated dimple by *n*th pulse in the burst expressed using Eqs. (9), (10), and (11):

12 $$V_{n} = \frac{\pi }{4}w^{2} \delta \ln \left( {\frac{{F_{n} }}{{F_{n}^{{{\text{th}}}} }}} \right)^{2} .$$

The peak laser fluence in the Gaussian beam in the pulse:

13 $$F_{{{\text{pulse}}}} = \frac{{2E_{{{\text{pulse}}}} }}{{\pi w^{2} }},$$

where *E*_pulse_ is the pulse energy of *n*th pulse in the burst. Ablation efficiency of *n*th pulse:

14 $$\eta_{n}^{{{\text{pulse}}}} = \frac{{V_{n} }}{{E_{{{\text{pulse}}}} }}.$$

By combining Eqs. (12), (13), and (14) we get the final expression ablation efficiency of *n*th pulse:

15 $$\eta_{n}^{{{\text{pulse}}}} = \frac{\delta }{{2F_{{{\text{pulse}}}} }}\ln^{2} \left( {\frac{{F_{n} }}{{F_{n}^{{{\text{th}}}} }}} \right).$$

From (15) we can also obtain ablation efficiency for (*n* + 1)th pulse:

16 $$\eta_{n + 1}^{{{\text{pulse}}}} = \frac{\delta }{{2F_{{{\text{pulse}}}} }}\ln^{2} \left( {\frac{{F_{n + 1} }}{{F_{n + 1}^{{{\text{th}}}} }}} \right).$$

Shielding of (*n* + 1)th pulse by attenuation of ablation cloud created by *n*th pulse expressed by Beer-Lambert-Bouguer absorption law:

17 $$F_{n + 1} = F_{{{\text{pulse}}}} \exp \left( { - K_{{{\text{eff}}}} C_{n} L_{n} } \right),$$

where *K*_eff_—effective shielding coefficient which takes into account attenuation by particles, vapor, and plasma generated by the previous pulse, *C*_*n*_ is the total concentration of shielding cloud (particles, vapor, and plasma), *L*_*n*_ is the averaged cloud thickness. The absorption coefficient of cloud can be expressed^54,61^:

18 $$\mu = \frac{4\pi \kappa }{\lambda },$$

where *κ* is the extinction coefficient (imaginary part of refractive index) and *λ* is the wavelength of irradiation. The mass attenuation coefficient (also known as the mass absorption coefficient) can be evaluated as^62,63^:

19 $$\frac{\mu }{{\rho_{{\text{m}}} }},$$

where *ρ*_m_ is the mass density of the cloud. The mass attenuation coefficient *µ*/*ρ*_m_ of liquid or solid copper can be used as effective shielding coefficient *K*_eff_ with some tolerance in our toy model since attenuation coefficient values of ablation cloud are not available in scientific literature.

From (16) and (17) we get an expression of ablation efficiency for (*n* + 1)th pulse:

20 $$\begin{aligned} \eta_{n + 1}^{{{\text{pulse}}}} & = \frac{\delta }{{2F_{{{\text{pulse}}}} }}\ln^{2} \left( {\frac{{F_{{{\text{pulse}}}} \exp \left( { - K_{{{\text{eff}}}} C_{n} L_{n} } \right)}}{{F_{n + 1}^{{{\text{th}}}} }}} \right) \\ & = \frac{\delta }{{2F_{{{\text{pulse}}}} }}\left( {\ln \left( {\frac{{F_{{{\text{pulse}}}} }}{{F_{n + 1}^{{{\text{th}}}} }}} \right) - K_{{{\text{eff}}}} C_{n} L_{n} } \right)^{2} . \\ \end{aligned}$$

In the (20) expression the product of concentration and the thickness of the cloud *C*_*n*_*L*_*n*_ is unknown. It depends on the known material and processing parameters like material density *ρ*, effective penetration depth *δ*, peak laser fluence *F*_pulse_*,* and the ablation efficiency of the previous pulse *η*_*n*_*.* The unknown parameters will be replaced by the known ones in the final equation.

The mass of ablation debris created by *n*th pulse, *C*_*n*_ is the total concentration of shielding cloud (particles, vapor, and plasma), *L*_*n*_ is the averaged cloud thickness. The mass *m*_*n*_ of ablation products created by *n*th pulse can be expressed:

21 $$m_{n} = \rho V_{n} = C_{n} L_{n} \frac{{{\uppi }D_{n}^{2} }}{4},$$

where *ρ* is the abated material density, *V*_*n*_ is the volume ablated by *n*th pulse described by Eq. (11), *C*_*n*_ is the total concentration of shielding cloud (particles, vapor, and plasma), *L*_*n*_ is the averaged cloud thickness and *D*_*n*_ cylindrical cloud diameter. Our initial assumption, suggesting that the cloud of ablation products adopts a cylindrical shape, may not be entirely accurate. Instead, it seems that the evolution of the cloud tends to favor a semi-spherical shape within the time scale of 15.5 ns, indicating that this approach might be more appropriate. In real conditions, the ablation cloud is the densest in the center and density decreases to the edges. However, the simplest way of defining plasma in the cylindrical shape with the diameter of the ablated area, certain depth and constant density not depending on the distance from the center. Here, because of simplified cylindrical shape we gain the mathematical advantage in the Beer–Bouguer–Labert exponential absorption equation, and we do not have complex variation within distance from the center, thus the fluence of the next pulse has still Gaussian transverse distribution, only with attenuated peak fluence value, and its lead to parabolic shape cavity ablation with smaller volume. With more realistic, semi-spherical cloud shape we would have mathematically much more complicated equations, and we would not be able to derive simplified recurrence relation equations. Therefore, we have made assumption in our toy model that ablation cloud has a cylindrical shape. The product of ablation cloud concentration and cloud depth can be expressed from Eq. (21):

22 $$C_{n} L_{n} = \frac{4\rho }{\pi }\frac{{V_{n} }}{{D_{n}^{2} }}.$$

The ratio between the volume of the dimple and the diameter squared can be expressed from Eqs. (10) and (12):

23 $$\frac{{V_{n} }}{{D_{n}^{2} }} = \frac{\pi }{8}\delta \ln \left( {\frac{{F_{n} }}{{F_{n}^{{{\text{th}}}} }}} \right).$$

From (22) and (23) we can get:

24 $$C_{n} L_{n} = \frac{\rho \delta }{{2}}\ln \left( {\frac{{F_{n} }}{{F_{n}^{{{\text{th}}}} }}} \right).$$

The Eq. (15) can be transformed as follows:

25 $$\ln \left( {\frac{{F_{n} }}{{F_{n}^{{{\text{th}}}} }}} \right) = \sqrt {\frac{{2F_{{{\text{pulse}}}} \eta_{n}^{{{\text{pulse}}}} }}{\delta }} .$$

From (24) and (25) we finally achieve an equation describing how the product of concentration and depth of the shielding cloud depends on the ablation efficiency of the previous pulse:

26 $$C_{n} L_{n} = \rho \sqrt {\frac{{F_{{{\text{pulse}}}} \delta \eta_{n}^{{{\text{pulse}}}} }}{2}} .$$

From Eqs. (20) and (26) finally, the new equation can be derived:

27 $$\eta_{n + 1}^{{{\text{pulse}}}} = \frac{\delta }{{2F_{{{\text{pulse}}}} }}\left( {\ln \left( {\frac{{F_{{{\text{pulse}}}} }}{{F_{n + 1}^{{{\text{th}}}} }}} \right) - K_{{{\text{eff}}}} \rho \sqrt {\frac{{\delta F_{{{\text{pulse}}}} \eta_{n}^{{{\text{pulse}}}} }}{2}} } \right)^{2} ,$$

this type of equation in mathematics is commonly called recurrence relation because the ablation efficiency *η*_*n*+1_ of (*n* + 1)th pulse is a function of efficiency *η*_*n*_ of *n*th pulse as *η*^pulse^_*n*+1_ = *f*(*η*^pulse^_*n*_), mathematically defined by Eq. (27). If the function *f* is not linear, the bistability may be observed.

The ablation efficiency of the burst *η*_burst_ can be evaluated as the average value of all ablation efficiencies for all pulses in the burst:

28 $$\eta_{N}^{{{\text{burst}}}} = \frac{1}{N}\sum\limits_{n = 1}^{N} {\eta_{n}^{{{\text{pulse}}}} } ,$$

where *N* is the number of the pulses in the burst (burst length), *n* is the number indicating pulse in the burst*.* The burst fluence *F*_burst_ can be computed by multiplying a single pulse fluence by the burst length as *F*_burst_ = *NF*_pulse_. The ablation threshold of (*n* + 1)th pulse dependence on the number of pulses *n*th applied before can be expressed as^44^:

29 $$F_{n + 1}^{{{\text{th}}}} = F_{\infty }^{{{\text{th}}}} + \left( {F_{1}^{{{\text{th}}}} - F_{\infty }^{{{\text{th}}}} } \right){\text{e}}^{ - kn} ,$$

*F*^th^_1_ is the single-shot threshold, the *F*^th^_∞_ multi-shot threshold, *k* is the empirical parameter in the exponent which characterizes the strength of incubation leading to an early reduction of the threshold.

### Spot diameter evaluation

Here we describe the measurement technique and its errors of spot diameter on the sample. The relationship between the crater diameter *D* and the peak laser fluence *F*_0_ at the center of the Gaussian beam may be expressed by Eq. (10), assuming that the laser beam has the Gaussian spatial beam profile^25,64^. The ablated craters on copper sample were characterized using optical microscopy. The diameters of the craters were measured using an optical microscope (Eclipse LV100, Nikon) utilizing a high-definition 5-megapixel CCD digital camera (DS-Fi1, Nikon) with a resolution of 2560 × 1920. The camera came with (NIS-Elements D, Nikon) image processing program, and (Digital Sight DS-U2, Nikon) controller. In the bright field mode, the objective (LU Plan Fluor 20x, Nikon) was utilized with a magnification factor of 20X and a numerical aperture (NA) = 0.5. A halogen light (LV-HL50PC, Nikon) was used to illuminate the specimen. The digital microscope image's pixel size variance was ≈0.2 µm, far less than the difference in crater size across craters ablated under identical experimental circumstances. As a result, five ablated craters' standard deviation was used to calculate measurement error. The Gaussian beam radiuses *w* were obtained from the line's slope using a semi-logarithmic plot of the diameter squared of the ablated area *D*_*n*_^2^ vs pulse energy *E*_pulse._ Equations (10) and (13) were utilized to compute the Gaussian beam radiuses based on the linear fit slopes. The average relative error of the beam radius measurements was < 2.7%. To adjust the laser spot radius on the copper sample, the sample's vertical location (*z*) was moved from 0.0 mm (focal position) to 5.3 mm.

### Fluence evaluation

The Eq. (13) was used to evaluate the peak laser fluence in pulse, which is inversely proportional to the square of the spot radius and directly proportional to the laser output power. The average laser power with a relative error of 0.7%, was measured using a power meter (Nova IΙ, Ophir) equipped with a thermal power sensor (30A-BB-18, Ophir). As mentioned before, the beam radius had a relative average error was less than 2.7%. A peak fluence in the pulse was evaluated with an average relative error of < 3.9%. The same error applies to burst fluence evaluations.

### Ablation rate and surface roughness evaluation

A stylus profiler (Dektak 150, Veeco) was utilized to measure the depth profiles of laser-ablated cavities. The measurement resolution was set at 0.1 μm in *x* direction, 1 μm in *y* direction, and 1 nm in *z* direction. In Fig. 1c, the example of the profile is displayed. Using information from the profiles, experimental values of the ablation efficiency *η*_exp_, which were calculated as the ablated volume per pulse *V*_pulse_ divided by the pulse energy *E*_pulse_ were retrieved in the following manner utilizing the volume of the ablated cavities:

30 $$\eta_{\exp } = \frac{{V_{{{\text{pulse}}}} }}{{E_{{{\text{pulse}}}} }} = \frac{\Delta x\Delta y\Delta z}{{E_{{{\text{burst}}}} }} = \frac{{v_{x} \Delta yh_{{\text{z}}} }}{{f_{{{\text{burst}}}} E_{{{\text{burst}}}} L}} = \frac{{v_{x} \Delta yh_{{\text{z}}} }}{PL},$$

where, Δ*x* = *v*_*x*_/*f*_burst_ is the distance between bursts in *x* direction, *v*_*x*_ is the beam scanning speed in the *x* direction, *f*_burst_ is the burst repetition rate, Δ*y* is the distance between scanned lines (hatch) in the *y* direction, Δ*z* = *h*_z_/*L* is ablated depth per scan, *h*_z_ is the ablated depth of the cavity in the *z* direction, *L* is the number of layers of beam scanning*, E*_burst_ is the burst energy defined by *E*_burst_ = *NE*_pulse_, *N* is the bust length, and *P* is the laser output power. The surface roughness *R*_a_ values were obtained directly from the laser-milled cavity bottom line profiles. The surface roughness of the cavity bottom was correlated with the ablation depth measurement errors. A cavity's depth measurement had an average relative inaccuracy of 2.5%. With an accuracy of 1.0%, a galvanometer scanner (Intelliscan 14, Scanlab) was used to control the beam scanning speed on sample and the and hatching distance^65,66^. As mentioned before the average laser power measurements had relative error of 0.7%. As a result, the experimental values of ablation efficiency had an average relative error less than 3.0%.

## Data Availability

Data underlying the results presented in this paper are not publicly available at this time but may be obtained from the authors upon reasonable request.
